# Zinc homeostasis dysregulation in elderly patients with acute ischemic stroke: a multidimensional analysis integrating metallothionein, FADS1 activity, oxidative stress, and DNA integrity markers

**DOI:** 10.3389/fneur.2026.1790962

**Published:** 2026-05-25

**Authors:** Jing Zhou, Qian Zhang, Chunmei Wang, Tingting Zhao, Tongtong Qiu, Guojie Xu, Cuiying Gu, Lihua Yu, Zhanyuan Yu

**Affiliations:** 1 Department of Geriatrics, Central Hospital of Qinghe County, Xingtai, Hebei, China; 2 Department of Pharmacy, Qinghe County Hospital of Traditional Chinese Medicine, Xingtai, Hebei, China; 3 Clinical Laboratory, Central Hospital of Qinghe County, Xingtai, Hebei, China; 4 Department of Pathology, Central Hospital of Qinghe County, Xingtai, Hebei, China

**Keywords:** 8-OHdG, acute ischemic stroke, biomarker panel, elderly, FADS1, inflammatory markers, metallothionein, oxidative stress

## Abstract

**Background:**

Zinc homeostasis dysregulation has been implicated in the pathophysiology of ischemic stroke, yet the integrated role of metallothionein (MT), fatty acid desaturase 1 (FADS1), and oxidative DNA damage in elderly ischemic stroke patients remains poorly understood. Objective: To comprehensively evaluate the association between zinc homeostasis dysregulation and acute ischemic stroke in elderly patients through a multidimensional analysis incorporating serum zinc, MT-1, FADS1 activity index, 8-hydroxy-2′-deoxyguanosine (8-OHdG), and inflammatory and oxidative stress markers.

**Methods:**

This retrospective case–control study enrolled 400 elderly participants (200 ischemic stroke patients and 200 sex-matched healthy controls) from Qinghe County Central Hospital between July 2024 and July 2025. Serum zinc, metallothionein-1 (MT-1), FADS1 activity index (arachidonic acid/linoleic acid ratio as a surrogate of Δ5-desaturase activity), 8-hydroxy-2′-deoxyguanosine (8-OHdG), and inflammatory and oxidative stress markers (high-sensitivity C-reactive protein [hs-CRP], interleukin-6 [IL-6], malondialdehyde [MDA], and superoxide dismutase [SOD]) were measured. Spearman correlation, univariate logistic regression, and receiver operating characteristic (ROC) curve analyses were performed.

**Results:**

Stroke patients exhibited significantly lower serum zinc (75.78 ± 20.39 vs. 97.94 ± 18.35 μg/dL, *p* < 0.001) and MT-1 levels (8.61 ± 3.29 vs. 12.85 ± 3.57 ng/mL, *p* < 0.001), elevated FADS1 activity index (0.50 ± 0.11 vs. 0.39 ± 0.09, *p* < 0.001), and increased 8-OHdG (7.96 ± 2.26 vs. 4.17 ± 1.31 ng/mL, *p* < 0.001) compared with controls. Inflammatory and oxidative stress markers were also significantly altered: hs-CRP (6.63 ± 5.66 vs. 2.56 ± 1.78 mg/L, *p* < 0.001), IL-6 (8.80 ± 5.44 vs. 3.80 ± 2.11 pg./mL, *p* < 0.001), MDA (6.07 ± 1.92 vs. 3.88 ± 1.11 nmol/mL, *p* < 0.001), and SOD (98.83 ± 24.85 vs. 127.83 ± 22.52 U/mL, *p* < 0.001). Serum zinc correlated positively with MT-1 (*r* = 0.236, *p* < 0.001) and negatively with 8-OHdG (*r* = −0.402, *p* < 0.001). ROC analysis demonstrated that the combined biomarker panel achieved superior discriminative performance (AUC = 0.970) compared with individual markers.

**Conclusion:**

Zinc homeostasis dysregulation, characterized by reduced circulating zinc and MT-1 levels alongside elevated FADS1 activity and oxidative DNA damage, is significantly associated with ischemic stroke in elderly patients. These findings suggest that a multidimensional biomarker panel integrating zinc metabolism, lipid signaling, and DNA integrity markers may serve as a useful tool for characterizing the pathophysiological profile of ischemic stroke in elderly patients and warrants further prospective validation for risk stratification.

## Introduction

Ischemic stroke, the most common subtype of stroke caused by cerebral arterial occlusion, remains a leading cause of mortality and long-term disability worldwide, with the elderly population bearing the greatest burden of disease (1–3). The Global Burden of Disease Study estimates that stroke accounts for approximately 5.5 million deaths annually, with individuals aged 65 years and older representing the majority of affected patients (4). Despite advances in acute interventional therapies, including intravenous thrombolysis and mechanical thrombectomy, the identification of modifiable risk factors and reliable biomarkers for stroke prediction remains a clinical priority (5).

Zinc, an essential trace element abundantly distributed in the central nervous system, plays critical roles in neurotransmission, synaptic plasticity, and neuroprotection (6–8). Under physiological conditions, intracellular zinc concentrations are tightly regulated by metallothioneins (MTs), a family of low-molecular-weight, cysteine-rich proteins that serve as zinc reservoirs and redox sensors (9–11). Emerging evidence suggests that disruption of zinc homeostasis contributes to cerebral ischemia–reperfusion injury through mechanisms involving oxidative stress, neuroinflammation, and autophagy dysregulation (12–14). Population-based studies have demonstrated an inverse association between serum zinc levels and ischemic stroke incidence, particularly among elderly women (15).

Beyond its role in metal homeostasis, MT-1 has garnered attention as a potential neuroprotective agent. Preclinical studies have shown that MT-1 overexpression reduces infarct volume and improves functional outcomes following experimental cerebral ischemia, possibly through attenuation of oxidative damage and inflammatory cascades (16–18). The clinical significance of circulating MT-1 levels as a biomarker for stroke risk or severity, however, remains incompletely characterized.

The fatty acid desaturase 1 (FADS1), also known as Δ5-desaturase, is a rate-limiting enzyme in polyunsaturated fatty acid (PUFA) metabolism, catalyzing the conversion of dihomo-*γ*-linolenic acid to arachidonic acid (AA). The serum or plasma AA/linoleic acid (LA) ratio has been widely used as a surrogate marker of FADS1 (Δ5-desaturase) activity (19–21). Mendelian randomization studies have identified FADS1 genetic variants as significant modulators of stroke risk, presumably through effects on circulating AA levels and downstream eicosanoid biosynthesis (22–24). Elevated FADS activity may promote a pro-inflammatory lipid milieu, potentially exacerbating atherosclerotic plaque vulnerability and thrombotic complications.

Oxidative stress represents a unifying pathophysiological mechanism linking zinc dyshomeostasis, lipid peroxidation, and cerebral ischemic injury (25). 8-Hydroxy-2′-deoxyguanosine (8-OHdG), a stable product of oxidative DNA damage, has been validated as a reliable biomarker of systemic oxidative burden and has demonstrated prognostic value in stroke outcomes (26–28). The relationship between 8-OHdG and zinc-related biomarkers in the context of elderly ischemic stroke, however, has not been systematically investigated.

Although individual associations between zinc deficiency, MT-1, FADS1 activity, and oxidative stress markers with ischemic stroke have been reported, no study has simultaneously examined these mechanistically interconnected biomarkers within a unified analytical framework in elderly ischemic stroke patients. The present study aimed to comprehensively evaluate the association between zinc homeostasis dysregulation and acute ischemic stroke in elderly patients through a multidimensional analysis incorporating serum zinc, MT-1, FADS1 activity index, 8-OHdG, and inflammatory and oxidative stress markers. We hypothesized that acute ischemic stroke patients would exhibit a distinct biomarker profile characterized by reduced zinc and MT-1 levels, elevated FADS1 activity, and increased oxidative DNA damage, and that a combined biomarker panel would provide superior discriminative performance compared with individual markers.

## Materials and methods

### Study design and population

This retrospective case–control study was conducted at Qinghe County Central Hospital, Hebei Province, China, between July 2024 and July 2025. The study protocol was approved by the Institutional Ethics Committee (Approval number: 2024003), and all participants or their legal representatives provided written informed consent in accordance with the Declaration of Helsinki.

A total of 400 participants were enrolled, comprising 200 patients with acute ischemic stroke (stroke group) and 200 sex-matched healthy controls (control group). Ischemic stroke was diagnosed according to World Health Organization criteria and confirmed by computed tomography or magnetic resonance imaging within 24 h of symptom onset. Stroke etiology was classified using the Trial of Org 10,172 in Acute Stroke Treatment (TOAST) criteria.

Inclusion criteria for stroke patients were: (1) age ≥60 years; (2) first-ever acute ischemic stroke confirmed by neuroimaging; (3) blood samples obtained within 72 h of symptom onset; and (4) complete clinical and laboratory data. Exclusion criteria included: (1) hemorrhagic stroke or transient ischemic attack; (2) history of zinc supplementation within 3 months; (3) active malignancy; (4) severe hepatic or renal dysfunction; (5) autoimmune disorders; and (6) recent infection or inflammatory disease.

Control subjects were recruited from community health screening programs and matched for sex. Controls underwent comprehensive medical evaluation to exclude cerebrovascular disease, cognitive impairment, or conditions potentially affecting zinc metabolism.

### Clinical assessment

Demographic and clinical data were collected through standardized case report forms. Stroke severity was assessed using the National Institutes of Health Stroke Scale (NIHSS) within 24 h of admission. Traditional vascular risk factors, including hypertension, diabetes mellitus, dyslipidemia, smoking, and alcohol consumption, were documented based on medical history, medication use, and standardized diagnostic criteria.

### Laboratory measurements

Fasting venous blood samples were collected within 72 h of stroke onset or during scheduled health evaluations for controls. Serum was separated by centrifugation at 3000 rpm for 10 min and stored at −80 °C until analysis.

Serum zinc concentrations were determined by inductively coupled plasma mass spectrometry (ICP-MS; Agilent 7,700x, Agilent Technologies, United States) with a coefficient of variation <5%. Serum MT-1 levels were measured using a commercially available enzyme-linked immunosorbent assay (ELISA) kit (Human MT-1 ELISA Kit, Abcam, United Kingdom; sensitivity: 0.1 ng/mL; intra-assay CV: 4.8%; inter-assay CV: 6.2%).

FADS1 activity was estimated using the serum AA/linoleic acid (LA) ratio as a surrogate marker of Δ5-desaturase activity, as previously described (29). Fatty acid profiles were analyzed by gas chromatography (GC-2010, Shimadzu, Japan). Serum 8-OHdG levels were quantified using a competitive ELISA kit (8-OHdG Check, Japan Institute for the Control of Aging, Japan; sensitivity: 0.5 ng/mL). Additional biomarkers included high-sensitivity C-reactive protein (hs-CRP) by immunoturbidimetry, interleukin-6 (IL-6) by ELISA, malondialdehyde (MDA) by thiobarbituric acid reactive substances assay, and superoxide dismutase (SOD) activity by colorimetric assay.

### Statistical analysis

Continuous variables were expressed as mean ± standard deviation (SD) and compared using Student’s t-test or Mann–Whitney U test as appropriate based on normality testing (Shapiro–Wilk test). Categorical variables were presented as frequencies (percentages) and compared using chi-square test. Correlations between continuous variables were assessed using Spearman’s rank correlation coefficient. Univariate logistic regression analysis was performed to estimate odds ratios (ORs) and 95% confidence intervals (CIs) for the association between each biomarker and stroke risk. Receiver operating characteristic (ROC) curve analysis was conducted to evaluate the diagnostic performance of individual biomarkers and the combined panel. The area under the curve (AUC), sensitivity, and specificity at the optimal threshold (determined by Youden’s index) were calculated. Subgroup analyses were performed stratifying by stroke severity (NIHSS ≤4: mild; NIHSS 5–15: moderate; NIHSS >15: severe). All statistical analyses were performed using Python 3.10 with SciPy and pandas libraries. A two-tailed *p* value <0.05 was considered statistically significant.

## Results

### Baseline characteristics

The stroke group was significantly older than the control group (71.44 ± 6.41 vs. 68.43 ± 5.51 years, *p* < 0.001; stroke group: range 60–85, median 71.0 years; control group: range 60–85, median 68.5 years). In the stroke group, male participants (*n* = 120) had a mean age of 71.26 ± 6.29 years and female participants (*n* = 80) had a mean age of 71.70 ± 6.62 years. In the control group, male participants (*n* = 114) had a mean age of 68.52 ± 5.60 years and female participants (*n* = 86) had a mean age of 68.31 ± 5.41 years. Sex distribution did not differ significantly between groups (male: 60.0% vs. 57.0%, *p* = 0.612). The significant age difference between groups was considered as a potential confounding factor in the interpretation of results. Among stroke patients, the mean NIHSS score at admission was 7.9 ± 2.5. Stroke severity was classified as mild (NIHSS ≤4, *n* = 16, 8.0%), moderate (NIHSS 5–15, *n* = 184, 92.0%), and severe (NIHSS >15, *n* = 0, 0%). The predominance of moderate-severity strokes reflects the clinical profile of our elderly cohort. The demographic and clinical characteristics of the study population are summarized in Table 1. As expected, traditional vascular risk factors were significantly more prevalent in stroke patients, including hypertension (71.0% vs. 37.5%, *p* < 0.001), diabetes mellitus (42.5% vs. 15.0%, *p* < 0.001), dyslipidemia (48.0% vs. 18.5%, *p* < 0.001), and smoking history (40.0% vs. 23.0%, *p* < 0.001). Stroke subtypes according to TOAST classification included large-artery atherosclerosis (33.5%), small-vessel occlusion (31.0%), cardioembolism (26.0%), and other determined or undetermined etiology (9.5%).

**Table 1:** Demographic and clinical characteristics of the study population.

Characteristic	Control (*n* = 200)	Stroke (*n* = 200)	*p* value
Age, years	68.43 ± 5.51	71.44 ± 6.41	<0.001
Male, *n* (%)	114 (57.0)	120 (60.0)	0.612
BMI, kg/m^2^	24.66 ± 2.90	25.77 ± 3.42	0.001
Hypertension, *n* (%)	75 (37.5)	142 (71.0)	<0.001
Diabetes mellitus, *n* (%)	30 (15.0)	85 (42.5)	<0.001
Dyslipidemia, *n* (%)	37 (18.5)	96 (48.0)	<0.001
Smoking history, *n* (%)	46 (23.0)	80 (40.0)	<0.001
Alcohol consumption, *n* (%)	33 (16.5)	59 (29.5)	0.003

Data are presented as mean ± SD or *n* (%). BMI, body mass index.

### Zinc homeostasis and related biomarkers

**Figure 1:**
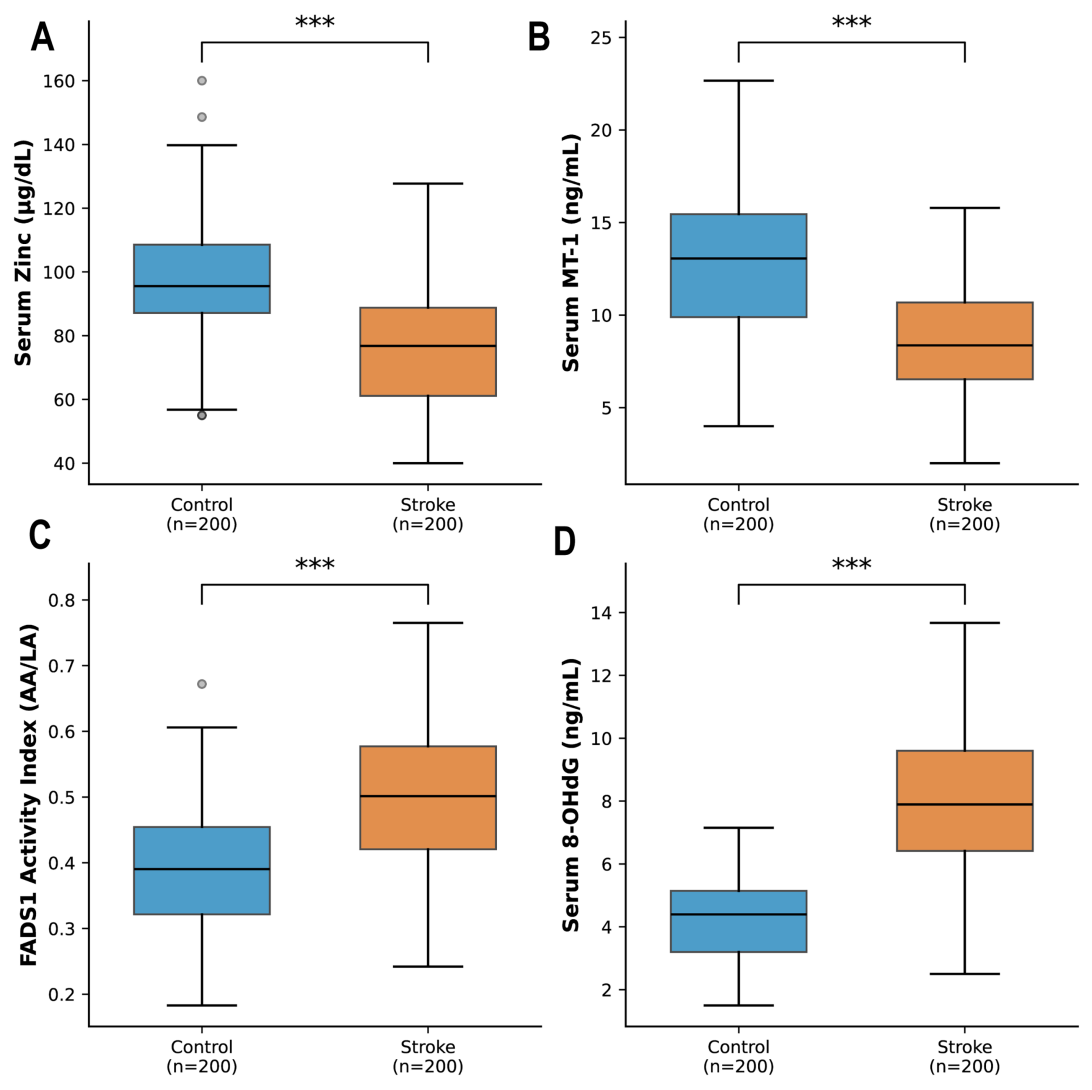
Comparison of zinc homeostasis biomarkers between control and stroke groups. Box plots showing the distribution of **(A)** serum zinc, **(B)** metallothionein-1 (MT-1), **(C)** FADS1 activity index (AA/LA ratio), and **(D)** 8-hydroxy-2′-deoxyguanosine (8-OHdG) levels in control subjects (blue, *n* = 200) and ischemic stroke patients (red, *n* = 200). Boxes represent the interquartile range (IQR), horizontal lines indicate the median, whiskers extend to 1.5 × IQR, and individual points represent outliers. ***p < 0.001.

Comparison of zinc homeostasis biomarkers between groups is presented in Table 2 and Figure 1. Stroke patients demonstrated significantly lower serum zinc levels compared with controls (75.78 ± 20.39 vs. 97.94 ± 18.35 μg/dL, *p* < 0.001). Similarly, serum MT-1 concentrations were markedly reduced in the stroke group (8.61 ± 3.29 vs. 12.85 ± 3.57 ng/mL, *p* < 0.001). In contrast, markers of altered lipid metabolism and oxidative stress were elevated in stroke patients. The FADS1 activity index (AA/LA ratio, reflecting Δ5-desaturase activity) was significantly higher in the stroke group (0.50 ± 0.11 vs. 0.39 ± 0.09, *p* < 0.001). The FADS2 index (GLA/LA ratio) was also elevated in stroke patients (0.03 ± 0.01 vs. 0.02 ± 0.01, *p* < 0.001). Serum 8-OHdG, reflecting oxidative DNA damage, was substantially elevated in stroke patients (7.96 ± 2.26 vs. 4.17 ± 1.31 ng/mL, *p* < 0.001). Inflammatory and additional oxidative stress markers corroborated these findings. hs-CRP (6.63 ± 5.66 vs. 2.56 ± 1.78 mg/L, *p* < 0.001), IL-6 (8.80 ± 5.44 vs. 3.80 ± 2.11 pg./mL, *p* < 0.001), and MDA (6.07 ± 1.92 vs. 3.88 ± 1.11 nmol/mL, *p* < 0.001) were all significantly higher in stroke patients, while SOD activity was reduced (98.83 ± 24.85 vs. 127.83 ± 22.52 U/mL, *p* < 0.001).

**Table 2:** Comparison of zinc homeostasis and related biomarkers.

Biomarker	Control (*n* = 200)	Stroke (*n* = 200)	*p* value
Serum zinc, μg/dL	97.94 ± 18.35	75.78 ± 20.39	<0.001
MT-1, ng/mL	12.85 ± 3.57	8.61 ± 3.29	<0.001
FADS1 index (AA/LA)	0.39 ± 0.09	0.50 ± 0.11	<0.001
FADS2 index (GLA/LA)	0.02 ± 0.01	0.03 ± 0.01	<0.001
8-OHdG, ng/mL	4.17 ± 1.31	7.96 ± 2.26	<0.001
hs-CRP, mg/L	2.56 ± 1.78	6.63 ± 5.66	<0.001
IL-6, pg./mL	3.80 ± 2.11	8.80 ± 5.44	<0.001
MDA, nmol/mL	3.88 ± 1.11	6.07 ± 1.92	<0.001
SOD, U/mL	127.83 ± 22.52	98.83 ± 24.85	<0.001

Data are presented as mean ± SD. MT-1, metallothionein-1; FADS, fatty acid desaturase; AA, arachidonic acid; LA, linoleic acid; GLA, gamma-linolenic acid; 8-OHdG, 8-hydroxy-2′-deoxyguanosine; hs-CRP, high-sensitivity C-reactive protein; IL-6, interleukin-6; MDA, malondialdehyde; SOD, superoxide dismutase.

### Correlation analysis

**Figure 2:**
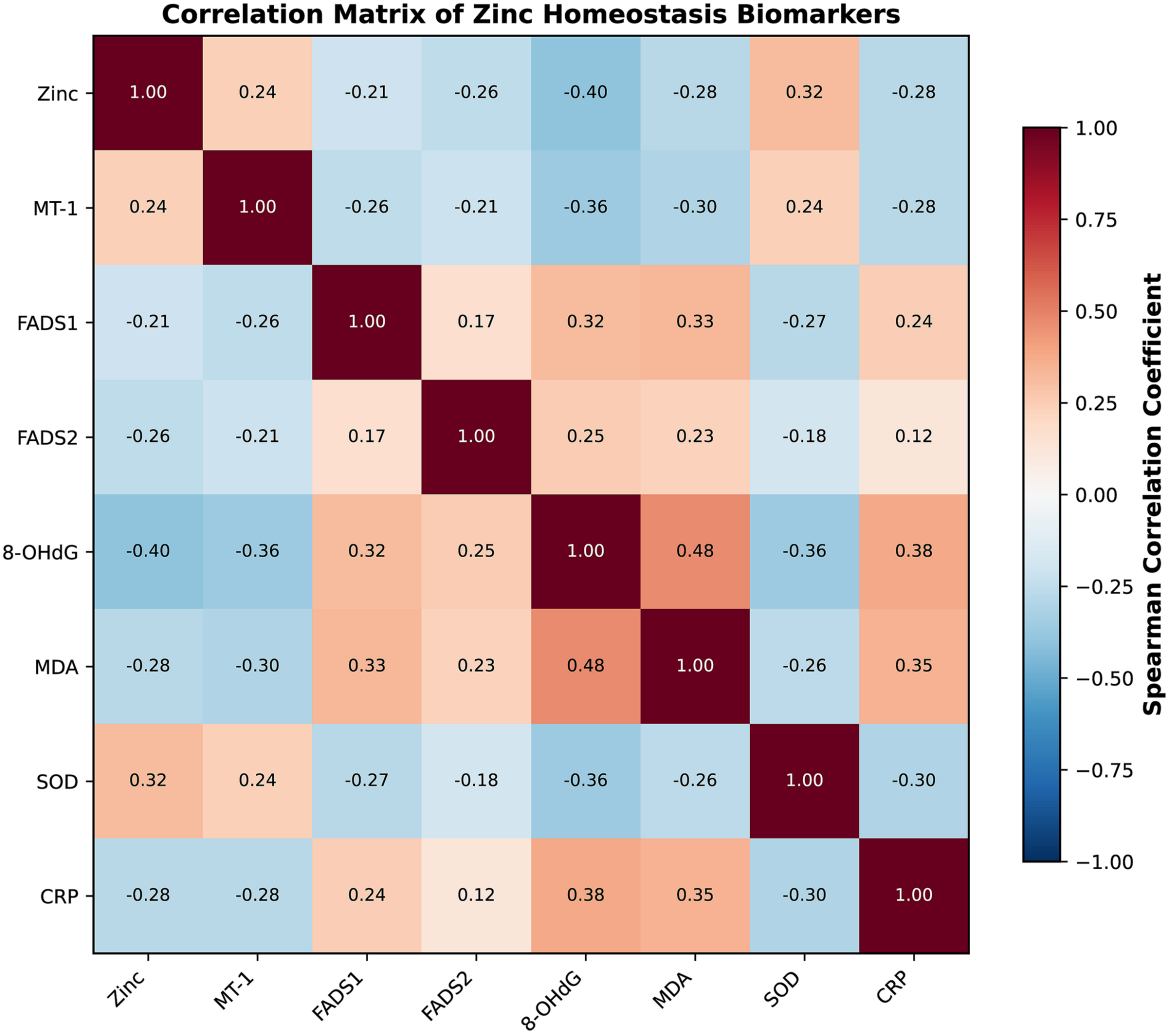
Correlation matrix of zinc homeostasis biomarkers. Heatmap displaying Spearman correlation coefficients among zinc homeostasis markers, inflammatory cytokines, and oxidative stress indicators. Color intensity indicates the strength of correlation, with positive correlations in red and negative correlations in blue. Correlation coefficients are displayed within each cell.

Spearman correlation analysis revealed significant associations among zinc homeostasis biomarkers (Figure 2). Serum zinc showed a positive correlation with MT-1 (*r* = 0.236, *p* < 0.001) and SOD (*r* = 0.238, *p* < 0.001), while demonstrating negative correlations with 8-OHdG (*r* = −0.402, *p* < 0.001) and FADS1 index (*r* = −0.211, *p* < 0.001). MT-1 was inversely correlated with 8-OHdG (*r* = −0.363, *p* < 0.001) and positively associated with SOD (*r* = 0.238, *p* < 0.001). The FADS1 index showed positive correlations with both 8-OHdG (*r* = 0.316, *p* < 0.001) and MDA (*r* = 0.333, *p* < 0.001), suggesting a link between enhanced desaturase activity and oxidative stress. Within the stroke subgroup, biomarker levels did not correlate significantly with NIHSS scores (zinc: *r* = 0.089, *p* = 0.210; MT-1: *r* = 0.032, *p* = 0.654; 8-OHdG: *r* = 0.020, *p* = 0.782), suggesting that these markers may reflect stroke susceptibility rather than acute lesion severity.

### Risk association analysis

**Figure 3:**
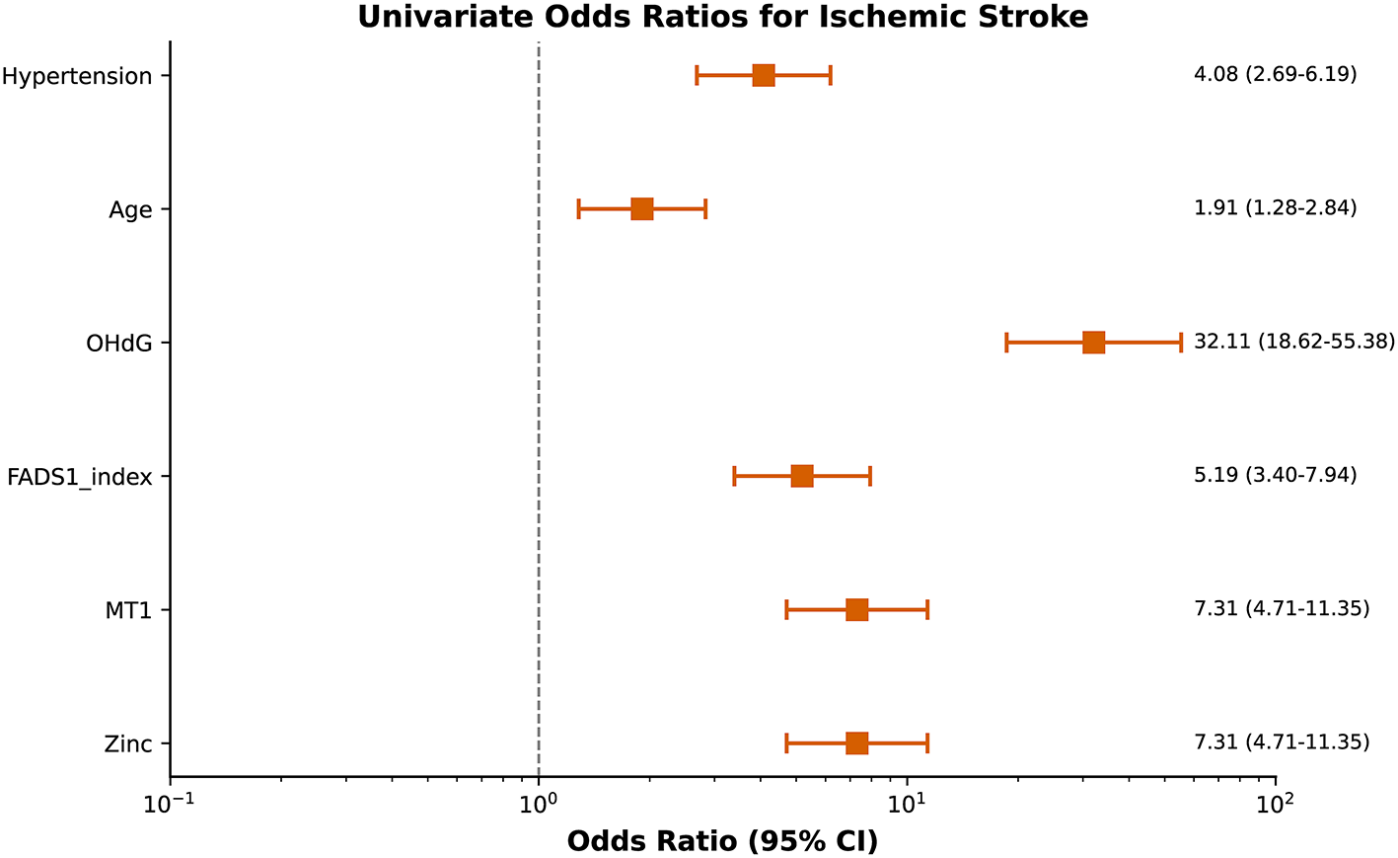
Forest plot of univariate odds ratios for ischemic stroke. Odds ratios (squares) and 95% confidence intervals (horizontal lines) for the association between each variable and stroke risk. Variables with OR >1 (red) indicate increased stroke risk; variables with OR<1 (blue) indicate decreased stroke risk. The vertical dashed line represents the null effect (OR = 1).

Univariate logistic regression analysis identified multiple biomarkers as significant predictors of stroke status (Table 3; Figure 3). Lower serum zinc (OR = 7.31, 95% CI: 4.71–11.35, *p* < 0.001) and MT-1 levels (OR = 7.31, 95% CI: 4.71–11.35, *p* < 0.001) were strongly associated with increased stroke odds. Elevated FADS1 index (OR = 5.19, 95% CI: 3.40–7.94, *p* < 0.001) and 8-OHdG (OR = 32.11, 95% CI: 18.62–55.38, *p* < 0.001) were also significant risk indicators. Among traditional risk factors, hypertension demonstrated the strongest association (OR = 4.08, 95% CI: 2.69–6.19, *p* < 0.001).

**Table 3:** Univariate logistic regression analysis for ischemic stroke risk.

Variable	OR	95% CI	*p* value
Low serum zinc (<85 μg/dL)	7.31	4.71–11.35	<0.001
Low MT-1 (<10 ng/mL)	7.31	4.71–11.35	<0.001
High FADS1 index (> 0.45)	5.19	3.40–7.94	<0.001
High 8-OHdG (>5.5 ng/mL)	32.11	18.62–55.38	< 0.001
Hypertension	4.08	2.69–6.19	<0.001
Diabetes mellitus	4.18	2.60–6.72	<0.001
Dyslipidemia	4.03	2.57–6.33	<0.001
Smoking history	2.24	1.45–3.45	<0.001

OR, odds ratio; CI, confidence interval; MT-1, metallothionein-1; FADS1, fatty acid desaturase 1; 8-OHdG, 8-hydroxy-2′-deoxyguanosine.

### Diagnostic performance

**Figure 4:**
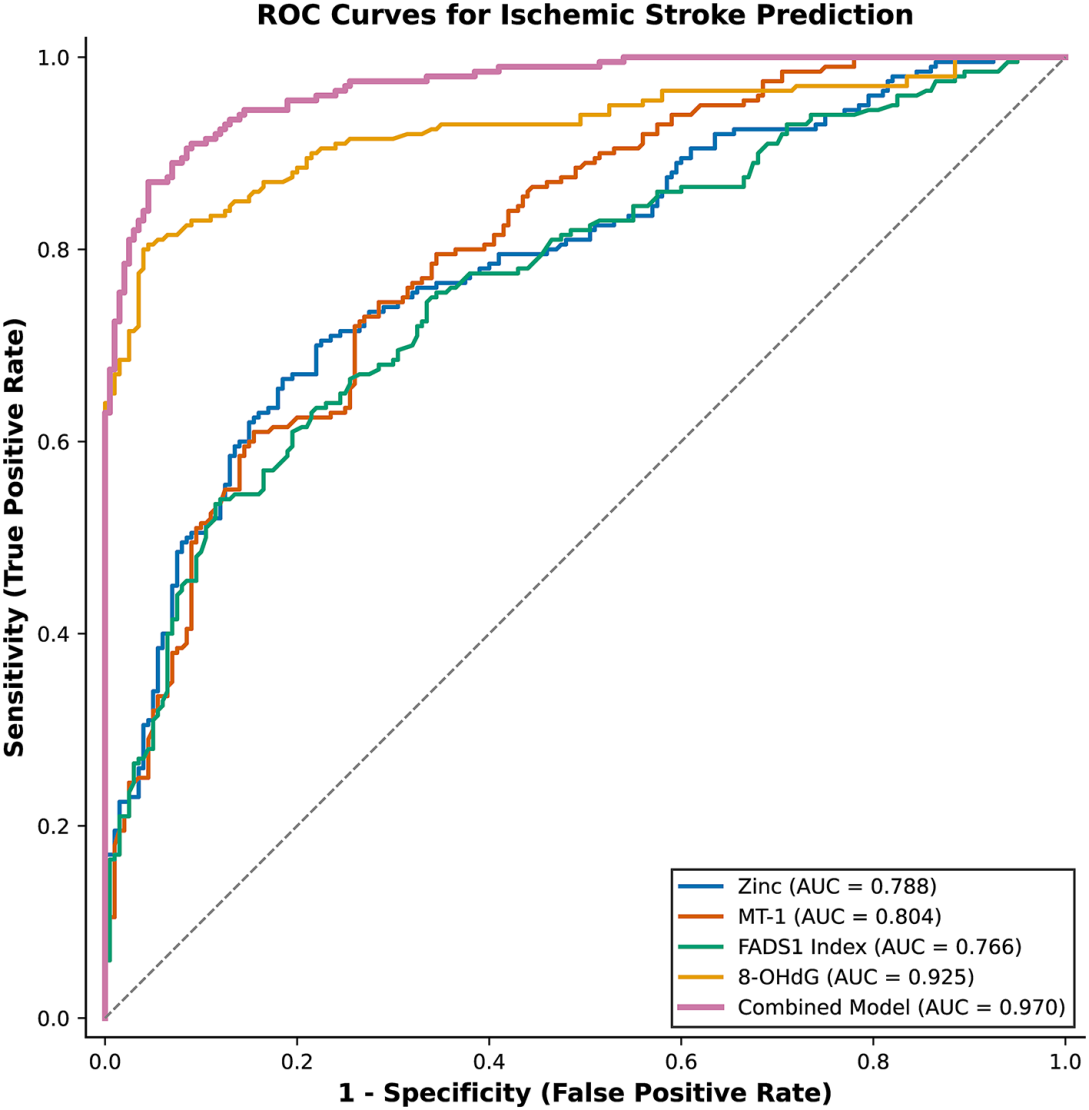
Receiver operating characteristic (ROC) curves for ischemic stroke prediction. ROC curves are shown for individual biomarkers (zinc, MT-1, FADS1 index, and 8-OHdG) and the combined biomarker panel. The area under the curve (AUC) for each marker is indicated in the legend. The diagonal dashed line represents the reference line (AUC = 0.5).

ROC curve analysis assessed the discriminative ability of individual biomarkers and the combined panel for distinguishing stroke patients from controls (Table 4; Figure 4). Among individual markers, 8-OHdG demonstrated the highest diagnostic accuracy (AUC = 0.925, sensitivity = 0.800, specificity = 0.960), followed by MT-1 (AUC = 0.804, sensitivity = 0.745, specificity = 0.715), serum zinc (AUC = 0.788, sensitivity = 0.665, specificity = 0.815), and FADS1 index (AUC = 0.766, sensitivity = 0.535, specificity = 0.885). The combined biomarker panel incorporating zinc, MT-1, FADS1 index, and 8-OHdG achieved superior diagnostic performance (AUC = 0.970, sensitivity = 0.870, specificity = 0.955), significantly outperforming any single marker. These findings support the clinical utility of a multidimensional approach to stroke risk assessment.

**Table 4:** Diagnostic performance of biomarkers for ischemic stroke.

Biomarker	AUC	Sensitivity	Specificity	95% CI
Serum zinc	0.788	0.665	0.815	0.748–0.828
MT-1	0.804	0.745	0.715	0.765–0.843
FADS1 index	0.766	0.535	0.885	0.724–0.808
8-OHdG	0.925	0.800	0.960	0.898–0.952
Combined panel	0.970	0.870	0.955	0.952–0.988

AUC, area under the curve; CI, confidence interval; MT-1, metallothionein-1; FADS1, fatty acid desaturase 1; 8-OHdG, 8-hydroxy-2′-deoxyguanosine. Combined panel includes zinc, MT-1, FADS1 index, and 8-OHdG.

## Discussion

The present study provides comprehensive evidence for zinc homeostasis dysregulation as a multifaceted pathophysiological feature of ischemic stroke in elderly patients. Our findings demonstrate that stroke patients exhibit a distinct biomarker profile characterized by reduced serum zinc and MT-1 levels, elevated FADS1 activity, and increased oxidative DNA damage. Importantly, the integration of these mechanistically related biomarkers into a combined panel substantially enhanced diagnostic performance, achieving an AUC of 0.970.

The observed reduction in serum zinc concentrations among stroke patients aligns with prior epidemiological evidence. The REGARDS study demonstrated an inverse association between serum zinc and ischemic stroke incidence, with individuals in the highest zinc quartile exhibiting 22% lower stroke risk compared with the lowest quartile (15). Our findings extend this observation to an elderly Chinese population and quantify the magnitude of zinc deficiency in acute stroke patients. The mean zinc level in our stroke cohort (75.78 μg/dL) was substantially lower than that of controls and approached the lower limit of the normal reference range (70–120 μg/dL). Notably, 78 of 200 stroke patients (39.0%) had zinc levels below 70 μg/dL, indicating a high prevalence of functionally relevant zinc deficiency in this population.

The parallel reduction in MT-1 levels provides mechanistic insight into zinc dyshomeostasis in stroke. Metallothioneins serve dual functions as zinc storage proteins and antioxidant enzymes, with their cysteine-rich structure enabling both metal chelation and free radical scavenging (30). Preclinical studies have demonstrated that MT-1 overexpression confers neuroprotection in experimental stroke models, reducing infarct volume by up to 42% (17). The positive correlation between zinc and MT-1 (r = 0.236) observed in our study suggests coordinated dysregulation of the zinc-MT axis, which may compromise both zinc bioavailability and antioxidant defense capacity. This finding is particularly relevant to understanding zinc homeostasis: MT-1 serves as the primary intracellular zinc buffer, and reduced MT-1 levels may impair the capacity to maintain zinc steady-state, leading to a vicious cycle of zinc depletion and diminished antioxidant protection. The inverse correlation between MT-1 and 8-OHdG (*r* = −0.363) further supports this interpretation, suggesting that MT-1 deficiency contributes to oxidative DNA damage accumulation.

A novel aspect of our investigation was the inclusion of the FADS1 activity index as a marker of altered lipid metabolism. FADS1 (Δ5-desaturase) catalyzes the conversion of dihomo-*γ*-linolenic acid to AA, a precursor of pro-inflammatory eicosanoids, and the serum AA/LA ratio specifically reflects FADS1 (Δ5-desaturase) activity rather than combined FADS1/2 activity (19). Elevated FADS1 activity, as reflected by the increased AA/LA ratio in stroke patients, may indicate enhanced production of pro-inflammatory and pro-thrombotic lipid mediators. Mendelian randomization studies have identified FADS1 variants as significant determinants of stroke risk (23), and our observational findings are consistent with a potential causal role for FADS-mediated lipid metabolism in cerebrovascular disease.

Our findings support a conceptual framework in which the measured biomarkers can be grouped into two mechanistically related clusters. First, the oxidative stress cluster: 8-OHdG, MDA, and SOD reflect different facets of oxidative damage and antioxidant defense. The positive correlation between FADS1 activity and both 8-OHdG (*r* = 0.316) and MDA (*r* = 0.333) suggests that enhanced desaturase activity promotes lipid peroxidation and oxidative DNA damage, while reduced SOD activity indicates compromised enzymatic antioxidant capacity. Second, the inflammatory cluster: the elevated AA/LA ratio (reflecting increased FADS1 activity) may drive pro-inflammatory eicosanoid biosynthesis, consistent with the observed elevations in hs-CRP and IL-6 in stroke patients. Arachidonic acid-derived prostaglandins and leukotrienes are well-established mediators of vascular inflammation and atherosclerotic plaque instability (31).

From a clinical perspective, the superior discriminative performance of the combined biomarker panel (AUC = 0.970) compared with individual markers supports the concept of ischemic stroke as a multifactorial disease involving interconnected pathophysiological pathways. However, it is important to note that because blood samples were collected within 72 h of symptom onset, these biomarkers reflect the acute-phase pathophysiological state rather than pre-stroke baseline levels. Therefore, the current findings should be interpreted as characterizing the multidimensional pathophysiological profile of acute ischemic stroke rather than as predictive or diagnostic biomarkers in the traditional sense. Future prospective studies with pre-stroke biomarker assessment are needed to determine whether these markers have predictive value for stroke occurrence or recurrence. Notably, the observed zinc deficiency and MT-1 reduction raise the possibility that correcting these abnormalities, such as through zinc supplementation, might reduce the probability of stroke recurrence, a hypothesis that warrants investigation in randomized controlled trials (32).

The lack of significant correlations between biomarker levels and NIHSS scores within the stroke subgroup (zinc: *r* = 0.089, *p* = 0.210; MT-1: *r* = 0.032, *p* = 0.654; 8-OHdG: *r* = 0.020, *p* = 0.782) suggests that these markers reflect underlying disease susceptibility rather than acute lesion severity. However, this interpretation should be considered with caution given that the majority of our patients had moderate-severity strokes (NIHSS 5–15, 92.0%), with very few mild cases (8.0%) and no severe cases, limiting the statistical power to detect severity-dependent biomarker changes. Future studies with a broader range of stroke severity are needed to clarify this relationship.

Several limitations warrant consideration. First, the retrospective design precludes causal inference regarding the temporal relationship between biomarker alterations and stroke occurrence. Prospective studies with pre-stroke biomarker assessment are needed to establish predictive validity. Second, blood samples were collected within 72 h of symptom onset, and acute-phase responses may have influenced biomarker levels; consequently, these biomarkers cannot be used for pre-stroke risk prediction or initial diagnosis, and serial measurements at multiple time points would provide a more comprehensive characterization of biomarker dynamics. Third, although the two groups were sex-matched, the stroke group was significantly older than the control group (71.44 ± 6.41 vs. 68.43 ± 5.51 years, *p* < 0.001), and the control group had a higher prevalence of certain comorbidities; this age difference and imperfect matching may confound the observed biomarker differences, and future studies should employ stricter age-matching or multivariate adjustment. Fourth, our study population was limited to a single center in northern China, potentially limiting generalizability to other ethnic or geographic populations. Fifth, we did not measure free zinc concentrations or assess cellular zinc distribution, which may provide additional mechanistic insights. Finally, the retrospective nature of the study precluded standardized dietary assessment, and dietary zinc intake could not be incorporated into the analysis.

This study demonstrates that zinc homeostasis dysregulation, characterized by reduced serum zinc and MT-1 levels alongside elevated FADS1 activity and oxidative DNA damage, is significantly associated with acute ischemic stroke in elderly patients. The integrated biomarker panel achieved excellent discriminative performance in distinguishing stroke patients from controls, suggesting potential utility for characterizing the pathophysiological profile of ischemic stroke. Future prospective studies are warranted to validate these findings, determine the predictive value of these biomarkers for stroke occurrence and recurrence, and explore the therapeutic potential of targeting the zinc-MT axis, including zinc supplementation, in cerebrovascular disease prevention.

## Data Availability

The original contributions presented in the study are included in the article/supplementary material, further inquiries can be directed to the corresponding author.

## References

[1] Feigin VL Brainin M Norrving B Martins S Sacco RL Hacke W . World stroke organization (WSO): global stroke fact sheet 2022. Int J Stroke. (2022) 17:18–29. doi: 10.1177/17474930211065917, 34986727

[2] Collaborators GBDS. Global, regional, and national burden of stroke and its risk factors, 1990-2019: a systematic analysis for the global burden of disease study 2019. Lancet Neurol. (2021) 20:795–820. doi: 10.1016/S1474-4422(21)00252-0 34487721 PMC8443449

[3] Martin SS Aday AW Almarzooq ZI Anderson CAM Arora P Avery CL . 2024 heart disease and stroke statistics: a report of US and global data from the American Heart Association. Circulation. (2024) 149:e347–913. doi: 10.1161/CIR.0000000000001209, 38264914 PMC12146881

[4] Zhou M Wang H Zeng X Yin P Zhu J Chen W . Mortality, morbidity, and risk factors in China and its provinces, 1990-2017: a systematic analysis for the global burden of disease study 2017. Lancet. (2019) 394:1145–58. doi: 10.1016/S0140-6736(19)30427-1, 31248666 PMC6891889

[5] Warner JJ Harrington RA Sacco RL Elkind MSV. Guidelines for the early Management of Patients with Acute Ischemic Stroke: 2019 update to the 2018 guidelines for the early Management of Acute Ischemic Stroke. Stroke. (2019) 50:3331–2. doi: 10.1161/STROKEAHA.119.027708, 31662117

[6] Yang X Li W Ding M Liu KJ Qi Z Zhao Y. Contribution of zinc accumulation to ischemic brain injury and its mechanisms about oxidative stress, inflammation, and autophagy: an update. Metallomics. (2024) 16:mfae012. doi: 10.1093/mtomcs/mfae012, 38419293

[7] Takeda A Fujii H Minamino T Tamano H. Intracellular Zn(2+) signaling in cognition. J Neurosci Res. (2014) 92:819–24. doi: 10.1002/jnr.23385, 24723300

[8] Qi Z Liu KJ. The interaction of zinc and the blood-brain barrier under physiological and ischemic conditions. Toxicol Appl Pharmacol. (2019) 364:114–9. doi: 10.1016/j.taap.2018.12.018, 30594689 PMC6331270

[9] Juarez-Rebollar D Rios C Nava-Ruiz C Mendez-Armenta M. Metallothionein in brain disorders. Oxidative Med Cell Longev. (2017) 2017:5828056. doi: 10.1155/2017/5828056, 29085556 PMC5632493

[10] Mohammed O Tufa A Gizaw ST. Emerging roles of Metallothioneins in human pathophysiology: a review. Health Sci Rep. (2025) 8:e71279. doi: 10.1002/hsr2.71279, 40994777 PMC12453973

[11] Ruttkay-Nedecky B Nejdl L Gumulec J Zitka O Masarik M Eckschlager T . The role of metallothionein in oxidative stress. Int J Mol Sci. (2013) 14:6044–66. doi: 10.3390/ijms14036044, 23502468 PMC3634463

[12] Li W Yang X Ding M Shi W Huang Y An Q . Zinc accumulation aggravates cerebral ischemia/reperfusion injury by promoting inflammation. Front Cell Neurosci. (2023) 17:1065873. doi: 10.3389/fncel.2023.1065873, 36970418 PMC10030816

[13] Qiu Z Liu X Li J Yin H Luo J Lin Z . Zinc alleviates stroke development through autophagy-mediated modulation of immune microenvironment. Front Immunol. (2025) 16:1666225. doi: 10.3389/fimmu.2025.1666225, 40969765 PMC12440758

[14] Liuzzi JP Guo L Yoo C Stewart TS. Zinc and autophagy. Biometals. (2014) 27:1087–96. doi: 10.1007/s10534-014-9773-0, 25012760 PMC4224969

[15] Mattern L Chen C McClure LA Brockman J Cushman M Judd S . Serum zinc levels and incidence of ischemic stroke: the reasons for geographic and racial differences in stroke study. Stroke. (2021) 52:3953–60. doi: 10.1161/strokeaha.120.033187, 34412513 PMC8608709

[16] van Lookeren Campagne M Thibodeaux H van Bruggen N Cairns B Gerlai R Palmer JT . Evidence for a protective role of metallothionein-1 in focal cerebral ischemia. Proc Natl Acad Sci USA. (1999) 96:12870–5. 10536015 10.1073/pnas.96.22.12870 PMC23139

[17] Smith HK Omura S Vital SA Becker F Senchenkova EY Kaur G . Metallothionein I as a direct link between therapeutic hematopoietic stem/progenitor cells and cerebral protection in stroke. FASEB J. (2018) 32:2381–94. doi: 10.1096/fj.201700746R, 29269399 PMC5901383

[18] Eidizadeh A Khajehalichalehshtari M Freyer D Trendelenburg G. Assessment of the therapeutic potential of Metallothionein-II application in focal cerebral ischemia in vitro and in vivo. PLoS One. (2015) 10:e0144035. doi: 10.1371/journal.pone.0144035, 26658636 PMC4682799

[19] Glaser C Heinrich J Koletzko B. Role of FADS1 and FADS2 polymorphisms in polyunsaturated fatty acid metabolism. Metabolism. (2010) 59:993–9. doi: 10.1016/j.metabol.2009.10.022, 20045144

[20] O'Neill CM Minihane AM. The impact of fatty acid desaturase genotype on fatty acid status and cardiovascular health in adults. Proc Nutr Soc. (2017) 76:64–75. doi: 10.1017/S0029665116000732, 27527582

[21] Park HG Engel MG Vogt-Lowell K Lawrence P Kothapalli KS Brenna JT. The role of fatty acid desaturase (FADS) genes in oleic acid metabolism: FADS1 Delta7 desaturates 11-20:1 to 7,11-20:2. Prostaglandins Leukot Essent Fat Acids. (2018) 128:21–5. doi: 10.1016/j.plefa.2017.11.004, 29413358 PMC5806126

[22] Yuan S Back M Bruzelius M Mason AM Burgess S Larsson S. Plasma phospholipid fatty acids, FADS1 and risk of 15 cardiovascular diseases: a Mendelian randomisation study. Nutrients. (2019) 11:3001. doi: 10.3390/nu11123001, 31817859 PMC6950527

[23] Zhang T Au Yeung SL Schooling CM. Associations of arachidonic acid synthesis with cardiovascular risk factors and relation to ischemic heart disease and stroke: a univariable and multivariable Mendelian randomization study. Nutrients. (2021) 13:1489. doi: 10.3390/nu13051489, 33924871 PMC8146807

[24] Martinelli N Girelli D Malerba G Guarini P Illig T Trabetti E . FADS genotypes and desaturase activity estimated by the ratio of arachidonic acid to linoleic acid are associated with inflammation and coronary artery disease. Am J Clin Nutr. (2008) 88:941–9. doi: 10.1093/ajcn/88.4.941, 18842780

[25] Chen S Li Q Shi H Li F Duan Y Guo Q. New insights into the role of mitochondrial dynamics in oxidative stress-induced diseases. Biomed Pharmacother. (2024) 178:117084. doi: 10.1016/j.biopha.2024.117084, 39088967

[26] Tugasworo DPA Kurnianto A Retnaningsih R Andhitara Y Ardhini R Budiman J. Malondialdehyde (MDA) and 8-hydroxy-2′-deoxyguanosine (8-OHdG) in ischemic stroke: a systematic review. Egypt J Neurol Psychiatry Neurosurg. (2023) 59:1–14. doi: 10.1186/s41983-023-00688-6

[27] Hsieh YW Lin KC Korivi M Lee TH Wu CY Wu KY. The reliability and predictive ability of a biomarker of oxidative DNA damage on functional outcomes after stroke rehabilitation. Int J Mol Sci. (2014) 15:6504–16. doi: 10.3390/ijms15046504, 24743892 PMC4013643

[28] Chen J Liu J Gu Z Fan J Lei S Zhang Q . Adherence to oxidative balance score is inversely associated with the prevalence of stroke: results from National Health and nutrition examination survey 1999-2018. Front Neurol. (2024) 15:1348011. doi: 10.3389/fneur.2024.1348011, 38638313 PMC11024455

[29] Huang L Chen Y Sun J Xu L. Exploring the correlation between dietary zinc intake and stroke risk in adults based on NHANES database. Neurol Res. (2024) 46:1113–21. doi: 10.1080/01616412.2024.2403858, 39510981

[30] Dai H Wang L Li L Huang Z Ye L. Metallothionein 1: a new spotlight on inflammatory diseases. Front Immunol. (2021) 12:739918. doi: 10.3389/fimmu.2021.739918, 34804020 PMC8602684

[31] Delgado-Martin S Martinez-Ruiz A. The role of ferroptosis as a regulator of oxidative stress in the pathogenesis of ischemic stroke. FEBS Lett. (2024) 598:2160–73. doi: 10.1002/1873-3468.14894, 38676284

[32] Liang Y Chen J Chen Y Tong Y Li L Xu Y . Advances in the detection of biomarkers for ischemic stroke. Front Neurol. (2025) 16:1488726. doi: 10.3389/fneur.2025.1488726, 40066310 PMC11891058

